# Prognostic relevance of positive urine markers in patients with negative cystoscopy during surveillance of bladder cancer

**DOI:** 10.1186/s12885-015-1089-0

**Published:** 2015-03-19

**Authors:** Tilman Todenhöfer, Jörg Hennenlotter, Philipp Guttenberg, Sarah Mohrhardt, Ursula Kuehs, Michael Esser, Stefan Aufderklamm, Simone Bier, Niklas Harland, Steffen Rausch, Georgios Gakis, Arnulf Stenzl, Christian Schwentner

**Affiliations:** 1 Department of Urology, Eberhard-Karls-University, Hoppe-Seyler-Str. 3, Tübingen, 72076 Germany; 2 Vancouver Prostate Centre, University of British Columbia, 2660 Oak Street, Vancouver, BC V3Z6H Canada

**Keywords:** Urine markers, Prediction, Recurrence, Risk, Surveillance, Anticipatory positive

## Abstract

**Background:**

The role of urine markers in the surveillance of patients with non-muscle invasive bladder cancer (NMIBC) is discussed extensively. In case of negative cystoscopy the additional prognostic value of these markers has not been clearly defined yet. The present study is the first systematic approach to directly compare the ability of a urine marker panel to predict the risk of recurrence and progression in bladder cancer (BC) patients with no evidence of relapse during surveillance for NMIBC.

**Methods:**

One hundred fourteen patients who underwent urine marker testing during surveillance for NMIBC and who had no evidence of BC recurrence were included. For all patients cytology, Fluorescence-in-situ-hybridization (FISH), immunocytology (uCyt+) and Nuclear matrix protein 22 enzyme-linked immunosorbent assay (NMP22) were performed. All patients completed at least 24 months of endoscopic and clinical follow-up of after inclusion.

**Results:**

Within 24 months of follow-up, 38 (33.0%) patients experienced disease recurrence and 11 (9.8%) progression. Recurrence rates in patients with positive vs. negative cytology, FISH, uCyt+ and NMP22 were 52.6% vs. 21.9% (HR = 3.9; 95% CI 1.75-9.2; p < 0.001), 47.6% vs. 25.0% (HR 2.7; 1.2-6.2; p = 0.01), 43.8% vs. 22.4% (HR 3.3; 1.5-7.6; p = 0.003) and 43.8% vs. 16.7% (HR 4.2; 1.7-10.8; p = 0.001). In patients with negative cytology, a positive NMP22 test was associated with a shorter time to recurrence (p = 0.01), whereas FISH or uCyt+ were not predictive of recurrence in these patients. In the group of patients with negative cytology and negative NMP22, only 13.5% and 5.4% developed recurrence and progression after 24 months.

**Conclusions:**

Patients with positive urine markers at time of negative cystoscopy are at increased risk of recurrence and progression. In patients with negative cytology, only NMP22 is predictive for recurrence. Patients with negative marker combinations including NMP22 harbour a low risk of recurrence. Therefore, the endoscopic follow-up regimen may be attenuated in this group of patients.

## Background

Patients with non-muscle invasive bladder cancer (NMIBC) harbour a significant risk of tumor recurrence and progression [1]. Several risk stratifications have been developed to predict the risk of recurrence or progression based on clinical and pathologic parameters [2]. However, these tools have only limited value in predicting the patients’ individual risk. Therefore, improved risk stratification is urgently needed. Due to the lack of reliable tools for prognosis of patients with NMIBC, the optimal follow-up of these patients is discussed controversially. White light cystoscopy remains the gold standard for surveillance of patients after NMIBC. The main limitations of cystoscopy are its limited sensitivity for flat lesions and its character as an invasive procedure potentially leading to significant discomfort for the patients [3,4]. Urine cytology is also recommended as standard in the follow-up of patients with NMIBC, as it is a non-invasive procedure with the potential to detect flat lesions not visible in cystoscopy [5]. However, its sensitivity is satisfactory only for high grade tumors or *carcinoma in situ*. Newer markers such as fluorescence-in-situ hybridization (FISH), immunocytology (uCyt+) or Nuclear matrix protein 22 (NMP22) have shown increased sensitivitity compared to cytology [6,7]. However, their specificity has been reported to be lower compared to cytology in most studies. For some of these markers, particularly the UroVysion FISH test previous studies have shown that a positive marker might precede visual or histologic detection of a tumor recurrence. Hence, some tests may be capable of detecting molecular changes associated with tumor recurrence earlier than cystoscopy [8,9]. The clinical implications and the optimal management of these patients with negative cystoscopy or biopsy and concomitantly positive markers remain to be defined.

To date only few data exist on the predictive values of multiple urine markers in this negative-cystoscopy setting. The present study is the first to address this issue concerning the four most widely available urine markers (Cytology, FISH, uCyt + and NMP22).

## Methods

### Patients and samples

114 patients (95 men and 19 women, median age 70, range 40-96) undergoing surveillance of NMIBC were enrolled. All patients had a negative cystocopy or, in case of suspicious findings, a negative histology at time of inclusion. Collection of urine samples was performed directly before cystoscopy. Upper tract imaging was performed in patients with positive cytology and/or FISH. In patients with suspicious or inconclusive findings in upper tract imaging, retrograde ureterorenoscopy was performed (n = 14, all negative). In patients with cytology highly suspicious for urothelial carcinoma (corresponding to categories IV + V of the Papanicolaou classification system), mapping biopsies were performed within four weeks of urine sampling (n = 9, all negative). Median time between last evidence of tumor and urine sampling & cystoscopy was six months (3-48). Written informed consent was obtained from all patients. The study was approved by the local ethics committee (Ethikkommission der Universität Tübingen, No. 400/2009A).

### Urine tests and diagnostic criteria

Urine samples of all patients were analyzed by cytology, FISH, uCyt + and NMP22). For cytology, Papanicoulaou staining was performed. Microscopic assessment was done according to the recommendations of the Papanicolaou Society of cytopathology [10]. The following features were assessed to identify malignant cells: papillary clusters of cells with eccentric nuclei, single cells with eccentric nuclei, an increased nuclear-to-cytoplasma ratio, irregular nuclear borders and coarse chromatin. Atypical urothelial cells and urothelial carcinoma cells (corresponding to categories III-V of the Papanicolaou classifiction system) were considered positive [11,12].

The UroVysion FISH assay was performed as previously described [13]. The following chromosomal patterns were required for a positive test: ≥4 out of 25 morphologically suspicious cells with ≥3 signals of at least two chromosomes 3, 7, 17 or ≥12 nuclei with homozygous loss of 9p21 [14]. Immunocytology (uCyt+) was preformed as described previously. The test was stated positive, if ≥1 cells show a clear (granulated) positive immunofluorescence signal of CEA or Mucin [15]. The NMP22 enzyme-linked immuno sorbet assay (ELISA) was performed according to the recommendations of the manufacturer and considered positive for values ≥10 IU/ml [16].

All patients underwent cystoscopy and biopsy or transurethral resection case of positive findings. Patients were considered positive for tumor if at least one suspicous area was observed during cystoscopy and malignancy was confirmed by subsequent histopathology.

### Follow-up

For all patients, an in-house cystoscopic follow-up of at least 24 months after urine sampling was available. Patients were followed up according to the recommendations of the European Association of Urology [1]. Recurrence was defined as histologically proven bladder cancer of any grade and stage within the follow-up of 24 months. Progression was defined as any increase in tumor stage or grade. Urine marker results obtained at later time points were not included into analysis.

### Statistics

Kaplan-Meier-curves were used to estimate times to recurrence and progression in patients with and without positive urine tests at time of cystoscopy. Log-rank test, univariate and multivariate Cox proportional hazard analyses were used to compare the risk of recurrence and progression in patients with and without positive marker(s). P-values ≤ 0.05 were considered significant. To compare rates of recurrence and progression between patients with different numbers of markers in a combination positive, the Cochrane Armitage test for trend was applied.

## Results

Patients’ characteristics are summarized in Table 1. During the follow-up of 24 months, 38 (33.0%) patients experienced disease relapse. The median time to recurrence was 11.5 months (3-24). 12 patients (10.5%) experienced disease progression during follow-up. Median time to progression was 11 months (3-24).

**Table 1:** **Patients’ characteristics**

Total number of patients, n	114
Age, years, Median (Range)	70 (40-96)
Gender, male/female	95/19
Recurrence within 24 months, n (%)	38 (33.0)
pT Stage of first recurrence within 24 months	
pTa	25 (65.8)
pT1	5 (13.1)
≥ pT2	3 (7.9)
Cis	11 (28.9)
G1	15 (39.5)
G2	9 (23.6)
G3	9 (23.6)
Interval between last bladder cancer episode and urine marker sampling, months, Median (Range)	6 (3-84)
pT/Grade last bladder cancer episode before urine marker sampling, n (%)	
pTa	74 (64.9)
pT1	28 (24.5)
Cis (pure)	12 (10.5)
Cis (concomitant)	11 (9.6)
G1	51 (44.7)
G2	35 (30.1)
G3	16 (14.0)
Highest pT/Grade in patient’s history before urine marker sampling, n (%)	
pTa	71 (62.3)
pT1	32 (27.3)
Cis (pure)	11 (9.6)
Cis (concomitant)	16 (14.0)
G1	47 (41.2)
G2	39 (34.2)
G3	17 (14.9)
Time to recurrence, months, Median (Range)	12.5 (3-24)
Patients developing progression, n (%)	13 (11.4)
Time to progression, months, Median (Range)	11 (3-24)

Cis = carcinoma in situ.

### Urine marker results

At the time of urine marker sampling cytology, FISH, uCyt+ and NMP22 ELISA were positive in 39 (34.2%), 42 (36.8%), 66 (57.8%) and 66 (57.8%) patients, respectively.

### Correlation of single urine markers with recurrence and progression

**Figure 1:**
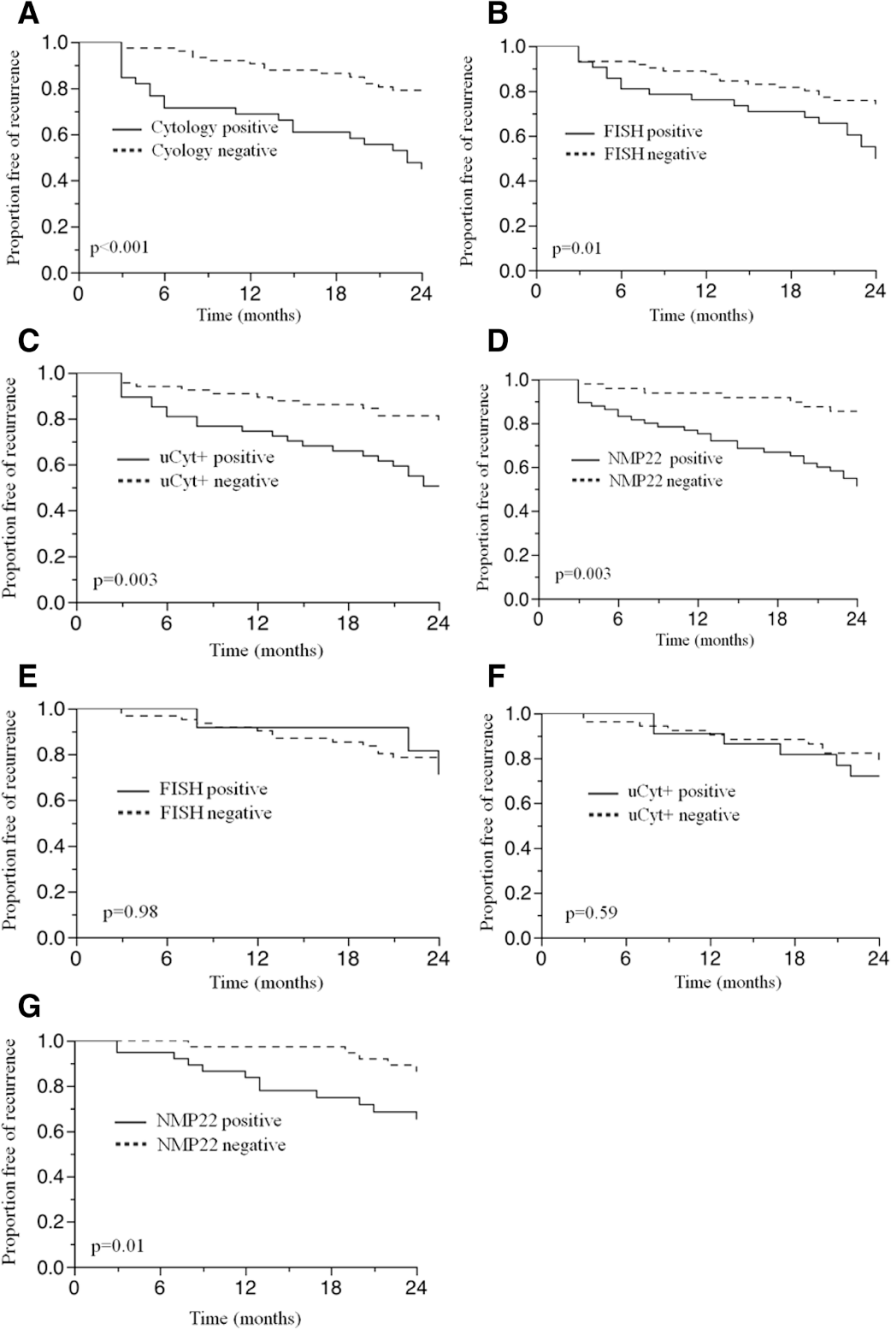
**Recurrence free survival in patients showing negative follow-up cystoscopy after nonmuscle invasive bladder cancer and single tests positive and negative for the whole cohort (A-D) and patients with negative cytology (E-G)**. FISH = Fluorescence in situ hybridization, NMP22 = Nuclear matrix protein 22, uCyt + = Immunocytology.

Rates of recurrence and progression and after 12 and 24 months in patients with negative and positive cytology, FISH, uCyt+ and NMP22 are summarized in Tables 2 and 3. Hazard ratios for recurrence and progression for patients with positive and negative markers are shown in Tables 2 and 3. Kaplan Meier analysis for recurrence and progression in patients with negative and positive markers are shown in Figure 1A-D. Single positive markers were associated with an increased risk for both recurrence and progression within 12 and 24 months. No significant differences were observed for rates of recurrences after 12 months in patients with negative and positive FISH and for rates of progression after 2 years in patients with negative and positive NMP22.

**Table 2:** **Prediction of recurrence by single urine markers in case of negative cystoscopy in the follow up of non muscle invasive bladder cancer**

Urine marker	Result	n	Rate of tumor recurrence in % (after 12 months)	Hazard ratio	p-value	Rate of tumor recurrence in % (after 24 months)	Hazard ratio	p-value
Cytology	-	75	9.3	4.3 (1.6-12.7)	.004	21.9	3.9 (1.75-9.2)	<.001
	+	39	30.7			52.6		
FISH	-	72	12.5	2.2 (0.8-6.0)	.12	25.0	3.3 (1.5-7.6)	.01
	+	42	23.8			47.6		
uCyt+	-	67	10.5	2.93 (1.2-8.0).	.03	22.4	2.7 (1.2-6.2)	.003
	+	66	25.5			43.8		
NMP22	-	48	6.3	4.79 (1.47-21.6)	.007	16.7	4.2 (1.7-10.8)	.001
	+	66	24.4			43.8		

FISH = Fluorescence in situ hybridization, uCyt + = Immunocytology, NMP22 = Nuclear matrix protein 22.

**Table 3:** **Prediction of progression by single urine markers in case of negative cystoscopy in the follow up of non muscle invasive bladder cancer**

Test	Result	n	Rate of tumor progression in % (12 months)	Hazard ratio	p-value	Rate of tumor progression in % (24 months)	Hazard ratio	p-value
Cytology	-	75	1.3	11.6 (1.7-226.1)	.008	4.0	7.2 (2.0-34.2)	<.001
	+	39	13.5			23.1		
FISH	-	72	1.4	9.72 (1.5-189.9)	.01	4.2	6.2 (1.7-29.7)	.004
	+	42	12.2			21.4		
uCyt+	-	67	1.5	8.3 (1.3-161.0)	.025	4.5	5.1 (1.4-23.8)	.01
	+	66	11.1			19.2		
NMP22	-	48	0	28×10^6^ (2.04-n.a.)	.009	6.2	2.4 (0.7-11.1)	.19
	+	66	9.2			13.6		

FISH = Fluorescence in situ hybridization, uCyt + = Immunocytology, NMP22 = Nuclear matrix protein 22.

As some of the recurrences detected in the first 12 months after initial cystoscopy might present no actual recurrences but tumors missed by initial cystoscopy, the role of urine markers to predict recurrence was determined separately for recurrences occurring later than one year after initial cystoscopy. Rates of recurrences and progression occurring between month 12-24 are shown in Table 4. Results of single markers remained predictive even for recurrences occurring later than 12 months whereas progression between month 12 and 24 only correlated with the results of cytology.

**Table 4:** **Prediction of recurrence and progression occurring between month 12-24 after urine sampling**

Test	Result	n	Rate of tumor recurrence in % (between month 12-24)	Hazard ratio	p-value	Rate of tumor progression in % (between month 12-24)	Hazard ratio	p-value
Cytology	-	68	14.7	2.89 (1.1-8.3)	.04	2.9	5.7 (1.1-43.4)	.04
	+	27	33.3			14.8		
FISH	-	63	14.3	2.72 (1.0-7.8)	.05	3.2	4.35 (0.8-32.8)	.08
	+	32	31.3			12.5		
uCyt+	-	60	13.3	2.9 (1.1-8.6)	.03	3.3	3.7 (0.7-28.1	.12
	+	35	31.4			11.4		
NMP22	-	45	11.1	3.1 (1.1-10.4	.03	6.7	1.11 (0.2-6.3)	.89
	+	50	28.0			6.0		

FISH = Fluorescence in situ hybridization, uCyt + = Immunocytology, NMP22 = Nuclear matrix protein 22.

### Anticipatory positive urine marker combinations

As cytology is the test most commonly used for surveillance of BC patients, we evaluated whether in patients with negative cytology, the results of other markers influence the risk of recurrence. Kaplan-Meier Analysis for patients with negative cytology and negative or positive FISH, uCyt+ and NMP22 are shown in Figure 1E-G. Recurrence free survival was significantly shorter for patients with positive vs. negative NMP22 at initial assessment (Figure 1G).

When performing various urine markers simultaneously, the risk of recurrence and progression may be influenced by the number of tests positive in a combination of two, three or four markers. Rates of recurrence and progression in patients with differing numbers of positive tests in 2-,3- or 4-test combinations are summarized in Table 5. The number of positive tests in the combinations was significantly associated both with recurrence and progression In patients having no marker positive in 2-test-combinations including NMP22, the rates of recurrence after 2 years were as low as 13.5% (NMP22 & cytology), 11.1% (NMP22 & FISH) and 9.4% (NMP22 & immunocytology). However, the proportion of patients with both tests negative in these combinations were only between 28.1% and 31.6%.

**Table 5:** **Risk of recurrence and progression according to the number of urine markers positive in 2-, 3-, and 4-marker combinations**

	Markers	Number of positive tests	n patients	Tumor recurrence (12 months) in %	p-value (0 vs. 1 vs. 2)	Tumor progression (12 months) in %	p-value (0 vs. 1 vs. 2)	Tumor recurrence (24 months) in %	p-value (0 vs. 1 vs. 2)	Tumor progression (24 months)in %	p-value (0 vs. 1 vs. 2)
**2-test-combinations**	Cytology & FISH	**0**	62	9.7	.01	1.6	.004	22.6	.001	3.3	<.001
		**1**	23	17.4		0		30.4		8.7	
		**2**	29	31.0		17.2		58.6		28.6	
	Cytology & uCyt+	**0**	53	9.4	.002	1.8	.003	20.8	<.001	3.8	<.001
		**1**	36	11.1		0		27.8		5.6	
		**2**	25	40.0		20.0		68.0		32.0	
	Cytology & NMP22	**0**	37	2.7	.004	0	.002	13.5	<.001	5.4	.004
		**1**	49	16.3		2.0		30.6		4.1	
		**2**	28	35.7		17.9		64.3		28.6	
	FISH & uCyt+	**0**	50	12.0	.02	2.0	.004	24.0	<.001	6.0	<.001
		**1**	39	10.3		0		23.1		0	
		**2**	25	36.0		20.0		68.0		36.0	
	FISH & NMP22	**0**	36	2.8	.008	0	.003	11.1	<.001	2.8	<.001
		**1**	48	20.8		2.1		37.5		8.3	
		**2**	30	26.7		16.7		53.3		23.3	
	uCyt + & NMP22	**0**	32	0	.001	0	.004	9.4	<.001	3.1	<.001
		**1**	51	19.6		1.9		33.3		7.8	
		**2**	31	29.0		16.1		58.1		22.6	
**3-test-combinations**	Cytology & FISH & uCYt	**0**	44	11.4	<.001	2.3	.001	22.7	<.001	4.6	<.001
		**1**	33	6.1		0		21.2		3.0	
		**2**	16	25.0		0		37.5		6.25	
		**3**	21	38.1		23.8		71.4		38.1	
	Cytology & FISH & NMP22	**0**	33	0	.001	0	.001	9.1	<.001	3.0	<.001
		**1**	36	22.2		2.8		38.9		5.6	
		**2**	24	12.5		0		25.0		8.3	
		**3**	21	38.1		23.8		71.4		33.3	
	Cytology & uCyt + & NMP22	**0**	37	2.7	<.001	0	.002	13.5	<.001	5.4	<.001
		**1**	38	15.8		2.6		31.6		2.6	
		**2**	11	18.2		0		27.3		9.1	
		**3**	28	35.7		17.8		64.3		28.6	
	FISH & uCyt + & NMP22	**0**	27	0	.002	0	.001	11.1	<.001	3.7	<.001
		**1**	37	18.9		2.7		27.0		5.4	
		**2**	32	15.6		0		37.5		6.25	
		**3**	18	38.9		27.8		72.2		38.9	
**4-test-combination**	Cytology & FISH & uCyt + & NMP22	**0**	26	0	<.001	0	<.001	11.5	<.001	3.9	<.001
		**1**	28	17.9		3.6		25.0		3.6	
		**2**	30	13.3		0		33.3		6.7	
		**3**	14	21.4		0		35.7		7.2	
		**4**	16	43.8		31.3		81.3		43.8	

p-values are given for Cochrane Armitage tests for trend. FISH = Fluorescence in situ hybridization, NMP22 = Nuclear matrix protein 22, uCyt + = Immunocytology.

Rates of recurrence and progression in patients with all urine tests positive vs. those with all tests negative were 81.3% vs. 11.5% (HR 33.2, 6.8-233.1, p < 0.001) and 43.8% vs. 3.8% (HR 19.4, 2.9-390.7, p = 0.001).

### Multivariable analysis

The risk of recurrence may be strongly influenced by other factors such as risk groups defined by the EAU [1]. As patients were included at different time points of follow-up, time interval between last tumor (proven by histopathology) and inclusion into the study might also strongly affect the results. Multivariate analyses controlling for results of cytology, interval between last tumor and inclusion into the study and risk groups (according to the guidelines of the EAU [1]) are shown in Table 6.

**Table 6:** **Mulitvariate analysis for prediction of recurrence adjusted for results of cytology, time interval between last NMIBC and urine sampling and European Organization for Research and Treatment of Cancer (EORTC) risk groups**

	Model 1 (Cytology)		Model 2 (Cytology & FISH)		Model 3 (Cytology& uCyt+)		Model 4 (Cytology & NMP22)	
	Hazard ratio	p	Hazard ratio	p	Hazard ratio	p	Hazard ratio	p
Cytology	2.8 (1.2-7.0)	.02	2.2 (0.8-6.3)	.13	1.9 (0.7-5.1)	.18	2.6 (1.0-6.5)	.04
FISH			1.6 (0.6-4.6)	.34				
uCyt+					3.23 (1.2-8.7)	.01		
NMP22							3.1 (1.2-8.6)	.01
Last tumor < vs. > 6 months	3.6 (1.5-8.9)	.001	3.8 (1.6-9.6)	.003	4.2 (1.7-11.0)	.002	3.0 (1.2-7.9)	.01
History of low/intermediate vs. high risk urothelial carcinoma	1.2 (0.5-2.9)	.83	1.1 (0.5-2.7)	.72	1.4 (0.5-3.5)	.51	1.2 (0.5-3.2)	.64

FISH = Fluorescence in situ hybridization, NMP22 = Nuclear matrix protein 22, uCyt+ = Immunocytology

## Discussion

The use of urine markers in the surveillance of patients with NMIBC has increased significantly in the last decade, although current guidelines give clear recommendations only for cytology. Urine markers are considered to be a valuable adjunct to cystoscopy [17,18]. However, the optimal work-up of patients with negative cystoscopy and positive urine markers - particularly for newer markers with decreased specificity compared to cytology - has not been clearly defined yet. It has been frequently discussed whether the presence of a positive marker in the absence of obvious changes in the bladder mucosa may indicate the presence of a non-visible tumor. Others even consider predicting the development of a tumor in the near future. Several reports indicate that anticipatory positive urine markers may indeed precede clinical tumor recurrence. The phenomenon of anticipatory positive markers has been firstly described by Steiner et al, who assessed the feasibility of microsatellite analysis in the follow up of 21 patients with NMIBC. In their cohort, two patients were positive for urine microsatellite analysis four and six months prior to cystoscopic detection of tumors [19]. A comparison of multiple urine markers and their combination has not been performed yet with regards to their anticipatory role. The aim of the present study was to compare the oncologic outcomes of patients with positive and negative cytology, FISH, uCyt+ and NMP22 at time of negative cystoscopy. Furthermore, we aimed to assess the impact of these markers on recurrence and progression when used in combinations.

In univariable analysis we observed for all markers to be highly predictive for recurrence and progression. Of note, patients with positive markers were at higher risk for recurrence even 1-2 years after urine marker testing and negative cystoscopy. This implies, that the tests might be able to display premalignant changes that are not visible macroscopically. Interestingly, in the group of patients having negative cytology, the only marker that was associated with increased risk of recurrence was NMP22. This indicates that both FISH and immunocytology markedly overlapped with cytology with regards to prediction. When combining urine markers, we observed that the more markers are positive in different combinations, the higher is the risk for recurrence and progression. For various combinations including NMP22, the risk of having disease recurrence and progression within 24 months was as low as 9.5-13.5% and 2.8-5.4% for patients with both tests negative (depending on the combination). Of note, a negative 4-test combination was not associated with a further decrease in the probability of recurrence and progression compared to the 2-test-combination with the highest negative predictive value (when considering recurrence as an event). Therefore, the use of more than two markers does not seem to provide additional benefit with regards to prediction of outcome.

The observation, that positive urine markers may precede visible tumor recurrence is in accordance with previous studies. In a cohort of 1114 patients tested by urine cytology, the anticipatory positive rate of cytology was 44% after a median time of 15 months [9]. Compared to our study, the rate of patients having a positive cytology but no positive histology within one year of follow-up was clearly lower. This finding may be related to the fact that cytology is strongly dependant on the cytopathologist [20].

UroVysion FISH has been observed to be positive before visible tumor recurrence in various studies. In a study by Yoder et al., 56 of 250 patients with atypical or negative cytology were positive for FISH and had no visible tumor recurrence at initial cystoscopy. In 35 of these patients (62.5%), tumor recurrence developed during the median follow-up of 23 months. Similar results were observed in a study including 68 patients under surveillance for NMIBC having negative cytology and cystoscopy at time of inclusion. During the median follow-up of 13.5 months, 45% of patients with positive UroVysion developed recurrence vs. 12.5% with normal UroVysion test. The proportion of patients with positive UroVysion at time of inclusion was considerably high (62.5%), given the fact that all were negative for cystoscopy at time of testing [21]. These findings are in contrast to our study, where in the group of patients with negative cytology, the rates of recurrence did not differ significantly between FISH positive and negative patients. This might be also caused by other criteria used for positive cytology. In a study from Italy 75 patients during follow-up of NMIBC were divided into patients with low molecular grade (changes in 9p21 or chromosome 3) and high-molecular grade (gains of chromosome 7 or 17) FISH results. Those patients with high molecular grade had a significantly shorter time to recurrence. In this study, no substratification was performed for cytology positive and negative patients [22]. However, the criteria used in this study were different compared to ours. It is broadly accepted that the interpretation criteria might strongly influence the prognostic value of UroVysion results. In a study including 138 patients with negative cystoscopy during follow-up for NMIBC, the UroVysion criteria proposed by the manufacturer were not predictive for recurrence. In contrast, criteria based on previous evidence suggesting that rare tetraploidic cells have a less strong diagnostic value than other aberrations, showed a significant association with disease recurrence [23,24]. Another test based on detection of genomic alterations which has been demonstrated to predict a later tumor recurrence is microsatellite analysis (MA). In a study by van Rhjin et al., MA was able to detect 75% of tumor recurrences. In the group of patients with positive MA and negative cystoscopy, 55% experienced recurrence within six months vs. 11.6% in patients with negative MA [25]. Similar results were obtained by van der Aa et al., who prospectitvely assessed the predictive value of MA in a longitudinal analysis for 458 series with persistent MA results. They observed, that patients with persistent positive MA analysis had a 83% risk of recurrence after two years vs. 22% in patients with persistent negative MA analysis. Positive MA series preceded recurrences by one to 24 months [26].

Analysis of FGFR3 mutations in voided urine samples of patients with history of FGFR3 mutant tumors was performed in a study by Zuiverloon et al. They observed, that that a single positive FGFR3 test was associated with a three times higher risk of a recurrence. In patients with consecutive FGFR3-positive urine samples the risk of developing a recurrence within 39 months was 90% [27].

For immunocytology, no report exists so far on anticipatory positive tests. In a study from Montreal including 109 patients during surveillance of BC, immunocytology results were correlated with tumor presence within 12 months [28]. However, the study, which showed a proportion of patients with tumor during 12 months of 76%, does not provide detailed information on when the recurrences occurred and how immunocytology predicted recurrences in patients with a negative cystoscopy at study inclusion. Similar to FISH, we observed no difference in recurrence free survival between patients with negative cytology and positive or negative immunocytology in our study. This might be due to a high percentage of patients (68.4%) showing an overlap of results of cytology and immunocytology.

Interestingly, NMP22 was the only marker being strongly predictive for recurrence in cytology negative patients. This might be explained by the different features covered by both tests. In our study, it was the test with the lowest concordance with cytology (43.0% of patients showed different results for cytology and NMP22). Such a strong correlation with risk of recurrence has not been observed in literature yet. However, the fact that NMP22 might be a test offering more additional information than other markers when used in combination with cytology has also been observed before [28,29]. Besides NMP22, other protein based urine markers have been also investigated for their potential in predicting recurrences in patients undergoing surveillance of NMIBC. In a study by Sanchez-Carbayo et al, patients under surveillance received serial testing for Urinary Bladder Cancer test (UBC), CYFRA-21-1 and NMP22. In the group of 65 patients with persistent negative markers during the study, only four patients developed recurrence within the one year follow-up of the study. The authors concluded, that urinary markers should be considered as adjuncts enabling individualized cystoscopy intervals during NMIBC surveillance [30].

The present study is the first study addressing the question, how combinations of multiple markers may help to improve prediction of recurrence and progression when assessed at time of negative cystoscopy. Although we observed that patients having all tests positive in a 4-marker-combination are at higher risk for recurrence than patients with less markers positive, the identification of patients with low risk of recurrence and progression was not improved significantly when using four instead of two markers. When discussing urine markers as potential tool to individualize cystoscopy intervals during BC surveillance, patients and urologists expect a high negative predictive value (NPV) for this test with regards to prediction of recurrence and/or progression. Therefore, the use of more than two markers has to be questioned in this context, as it does not seem to improve the NPV compared to the 2-test-combinations with high NPV. Furthermore, the use of multiple markers is associated with a significant increase in costs.

The limitations of our study include the size and the heterogeneity of the cohort. Although we controlled for this bias in multivariate analysis, the inclusion of patients with different intervals between last evidence of tumor and urine marker testing might have influenced the results. Furthermore, we cannot exclude that the results of the urine markers influenced cystoscopy intervals as treating urologist were not blinded to the test results. However, the inclusion of patients with a follow-up of at least 24 months and the performance of tests which took the presence of tumor within 24 months as an endpoint (regardless of the time to recurrence) may partially control for this bias. As only a part of the patients underwent mapping biopsies during cystoscopy, it cannot be completely ruled out that carcinoma in situ was present at time of initial cystoscopic assessment. Moreover, the sensitivity of upper tract imaging for detection of upper urinary tract urothelial carcinoma is limited. To exclude presence of upper tract tumors, a ureteroscopic assessment of all patients would have been necessary. However, none of the patients presented clinical symptoms of upper tract urothelial carcinoma within the follow-up of two years.

## Conclusions

The present study shows that patients with positive cytology, uCyt+, FISH or NMP22 test at time of negative cystoscopy exhibit a shorter time to disease recurrence and progression. For the first time, NMP22 is identified as a predictor of recurrence and progression in patients with negative cystoscopy and cytology. Negative combinations of NMP22 and a second urine marker are associated with a low risk of recurrence within two years. The endoscopic follow-up regimen may be therefore attenuated in patients with persistent negative results of these markers.
